# Employing a Monte Carlo Algorithm in Newton-Type Methods for Restricted Maximum Likelihood Estimation of Genetic Parameters

**DOI:** 10.1371/journal.pone.0080821

**Published:** 2013-12-10

**Authors:** Kaarina Matilainen, Esa A. Mäntysaari, Martin H. Lidauer, Ismo Strandén, Robin Thompson

**Affiliations:** 1 Biotechnology and Food Research, MTT Agrifood Research Finland, Jokioinen, Finland; 2 Biomathematics and Bioinformatics Department, Rothamsted Research, Harpenden, United Kingdom; Pennsylvania State University, United States of America

## Abstract

Estimation of variance components by Monte Carlo (MC) expectation maximization (EM) restricted maximum likelihood (REML) is computationally efficient for large data sets and complex linear mixed effects models. However, efficiency may be lost due to the need for a large number of iterations of the EM algorithm. To decrease the computing time we explored the use of faster converging Newton-type algorithms within MC REML implementations. The implemented algorithms were: MC Newton-Raphson (NR), where the information matrix was generated via sampling; MC average information(AI), where the information was computed as an average of observed and expected information; and MC Broyden's method, where the zero of the gradient was searched using a quasi-Newton-type algorithm. Performance of these algorithms was evaluated using simulated data. The final estimates were in good agreement with corresponding analytical ones. MC NR REML and MC AI REML enhanced convergence compared to MC EM REML and gave standard errors for the estimates as a by-product. MC NR REML required a larger number of MC samples, while each MC AI REML iteration demanded extra solving of mixed model equations by the number of parameters to be estimated. MC Broyden's method required the largest number of MC samples with our small data and did not give standard errors for the parameters directly. We studied the performance of three different convergence criteria for the MC AI REML algorithm. Our results indicate the importance of defining a suitable convergence criterion and critical value in order to obtain an efficient Newton-type method utilizing a MC algorithm. Overall, use of a MC algorithm with Newton-type methods proved feasible and the results encourage testing of these methods with different kinds of large-scale problem settings.

## Introduction

Estimation of variance components (VC) by restricted maximum likelihood (REML) [1] via a Monte Carlo (MC) expectation maximization (EM) algorithm has proven a computationally attractive choice for large data sets and complex linear mixed effects models [2]–[4]. In such cases, it is often impossible to calculate the exact inverse of the coefficient matrix using direct methods, but it can be estimated by MC sampling methods instead. Although the idea of MC EM REML is simple, its convergence is slow, like typical for the EM algorithm. There are different ways to enhance the convergence. One possibility is to use observed information obtained by Louis' method [5], which also gives standard errors for the estimates. The MC technique can be adapted to Louis's method as well [6]. Other possibilities include Aitken's acceleration and quasi-Newton EM acceleration, as used in [7] and discussed, e.g., in [8]. However, both Louis' method and the acceleration methods require complicated calculations which may be difficult with the large-scale problems often occurring in animal breeding evaluations.

Newton-type methods are based on second derivatives and reach fast convergence in the neighbourhood of the maximum. Second derivatives with respect to all the parameters yield the information matrix, which can be used to calculate standard errors for the parameters. The Newton-Raphson (NR) method is based on the observed information matrix while Fisher's scoring uses the expected information matrix. Other Newton-type methods include average information (AI) REML, which utilizes the average of the observed and expected information matrices [9]. This is currently the most common VC estimation method used in animal breeding. Quasi-Newton methods [10], which rely on approximation of second derivatives based on the direction of the most recent step, have also been suggested and used, e.g [11]. These methods usually result in faster convergence compared to linear methods but slower convergence compared to Newton-type methods because the information matrix is replaced by an approximation.

MC techniques are useful for analyses involving complex likelihoods. Thus, the MC method has been used in the NR algorithm for generalized linear mixed effects models, e.g., by Kuk and Cheng [12], and for incomplete data, e.g., by Gauderman and Navidi [13]. More complicated models related to these examples require simulations from the conditional distribution of the missing data given the observed data with methods like Gibbs sampling. However, the problem in animal breeding is not necessarily the complexity of the model, but rather the need to analyze large-scale data sets to obtain sufficiently accurate genetic parameter estimates. In such cases, the simple sampling method presented in García-Corts et al. [14] has shown to be practical for VC estimation in linear mixed effects models by MC EM REML [4]. Its use is also possible in Newton-type methods.

The aim of this study is to compare MC algorithms in different Newton-type methods for VC estimation of linear mixed effects models. We first introduce the AI REML and Broyden's method with MC. These methods are then compared with a sampling-based NR method (MC NR REML), where a simple approximation of second derivatives is possible from independent and identically distributed samples. Finally, we evaluate the performance of these Newton-type methods using the MC algorithm in an analysis of simulated example data.

## Materials and Methods

### Model

Consider a bivariate linear mixed effects model
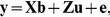
(1)where **y** is a vector of observations, **b** is a vector of fixed effects, **u** is a vector of random effects, **e** is a vector of random error terms or residuals, and **X** and **Z** are design matrices for fixed and random effects, respectively. Assume that 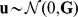 has a covariance structure 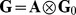, where **A** is a numerator relationship matrix and 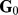 is a 2×2 covariance matrix. Similarly, 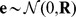, where 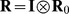 and 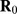 is a 2×2 covariance matrix. Thus, 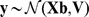, where 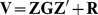. We assume that either both or no traits are observed.

### Methods

Let the parameter vector of covariances be 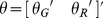, where 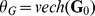, 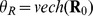 and *vech* is an operator changing unique elements of the matrix argument into vector form. In our case, *θ* is a 6×1 vector which contains three unique elements from both the random effect and residual covariance matrices. Newton-type methods rely on first and second derivatives of the REML likelihood function *L*(*θ*) with respect to *θ*. For example, the NR algorithm uses the observed information matrix
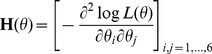
(2)and the gradient vector
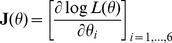
(3)in calculating new estimates of parameters 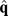 at iteration round *k*:
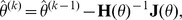
where information matrix 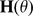 and gradient vector 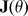 are computed at current VC estimate 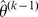.

First derivatives of the REML log-likelihood 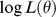 with respect to elements in 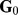 or 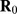 can be considered simultaneously [13]. Thus, 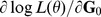 can be written as a 2×2 matrix which has diagonal elements 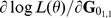 and 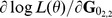 and off-diagonal elements 
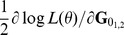
 and 
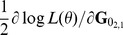
:
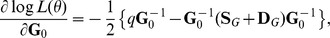
where *q* is the number of levels in random effect **u**, and 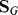 and 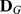 are 2×2 matrices with elements 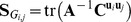 and 
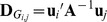
, respectively. Here 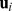 is a subvector of **u** corresponding to the 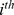 trait in the model, and 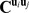 is the part of the inverse of the coefficient matrix of the mixed model equations (MME) corresponding to 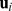 and 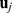, 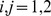. Similarly,
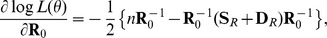
where *n* is the number of observations, and 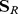 and 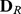 are 2×2 matrices with elements 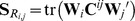 and 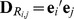. Now 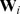 is a submatrix of 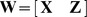 and 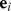 is a subvector of **e** corresponding to the 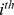 trait, and 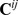 is the part of the inverse of the coefficient matrix of MME corresponding to traits *i* and *j*.

Matrices 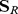 and 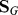 are difficult to compute for large data sets and complex models because they require elements of **C**, the inverse of the coefficient matrix of MME. These matrices can be approximated by simulating *s* MC samples of data, i.e., 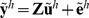, 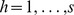 [14] where 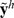 is a vector of MC simulated observations at MC sample *h*, and 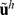 and 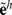 are simulated from their assumed normal density models using current values of the variance parameters. When the full model (1) is fitted, i.e., when MME are solved using the simulated data to obtain estimates 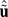, element (i,j) in 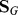 can be approximated by method 1 or 2 in García-Corts et al. [16]:
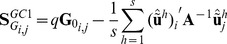
(4)or
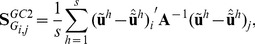
(5)respectively. These formulas are also convenient for multivariate models, as shown in Matilainen et al. [4]. An increase in MC sample size *s* will give more accurate estimates of the **C** and 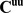 matrices and, subsequently, more accurate estimates of the gradient.

Elements in the observed information matrix (2) require more complex calculations than those needed to calculate the gradient vector (3). Approximations are used here to avoid calculations of exact second derivatives. In the following section we present three methods applying the MC sampling scheme. The first method, which is named MC NR REML, is based on calculation of the observed information matrix by sampling. The second method, named MC AI REML, uses MC sampling in the AI REML algorithm. Finally, the third MC sampling method, named MC BM REML, is based on Broyden's method.

MC NR REML. By definition, the expected information matrix at convergence is
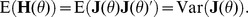
Use of the MC algorithm with independent and identically distributed samples enables approximation of the information matrix by the variances of the gradients over the samples within each NR REML round. Note, however, that (4) needs to be used to compute the sampling variance of the gradients, because (5) only gives the variances of prediction error variances. Now, the information matrix is approximated by
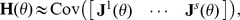
where
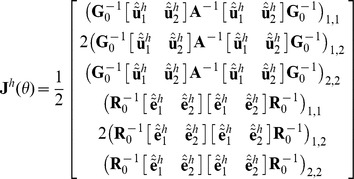
is a gradient vector calculated based on sample *h*, 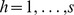. For a *s*×6 matrix **J**, 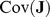 returns a 6×6 matrix, where the diagonal has variance within each column in **J**, and the off-diagonals contain the covariances between each two-column combinations in **J**.

MC AI REML. Johnson and Thompson [17] and Gilmour et al. [9] presented AI REML noting that computation of the average of the observed and expected information matrices is easier than of either of the components:
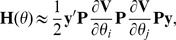
where
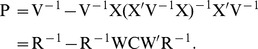
Define 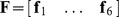, where 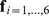 is
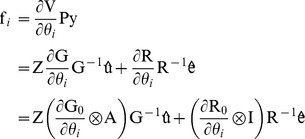
Then
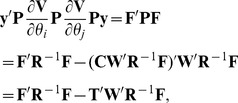
where
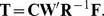
Hence, in MC AI REML, MC sampling is needed only for estimation of first derivatives in **F**, while the average information can be calculated based on current VC estimates. However, the algorithm requires additional computations to form **F**, which necessitates solving **T** from the MME with data replaced by 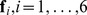. Thus, MME needs to be solved for each VC parameter.

MC BM REML. Broyden's method is a quasi-Newton method for numerical solution of non-linear equations [18]. It is a generalization of the secant method to multiple dimensions. Broyden's method updates the inverse of the information matrix (instead of the information matrix itself) within each round:
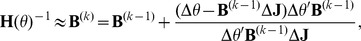
where
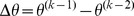


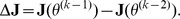
Instead of using true gradients 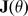 for both the update of the inverse information matrix and the update of new estimates, we used the round-to-round changes in the EM estimates [19]:
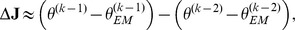
where 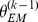 is a vector of EM REML solutions computed from the estimates from round 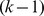. Apart from scaling, they are relative to the original gradients. In the beginning, 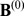 is identity matrix **I**. The first update of the inverse of the information matrix is made at round *k* = 2 based on estimates from the first round *k* = 1 and initial values at *k* = 0.

This method leads to superlinear convergence although sequence 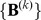 does not converge to the observed information matrix at the maximum [20]. Like with MC AI REML, MC sampling is not used to estimate the information matrix directly. Instead, the sampling variation comes in through the gradients which are used to update the inverse of the information matrix within each round.

### Analysis of test data

For this study we simulated a small data set which mimics a typical set-up in dairy cattle breeding. The two study traits resembled 305-day milk and fat yield records in 20 herds. The base generation comprised 146 unrelated sires, each of which had 1 to 10 daughters with unknown and unrelated dams. Each daughter had one observation of the two traits, and the data contained 569 observations for both traits. The pedigree comprised a total of 715 animals. Fixed herd effects and random genetic animal effects were included in the study model. Table 1 shows genetic and residual VC in 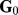 and 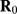 used to simulate 305-day milk and fat records. The simulation of observation was based on the assumed linear mixed effects model and VCs.

**Table 1:** Variance components used for the simulation, initial values used for the analyses and estimates by analytical EM REML.

	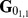	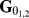	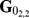	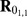	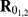	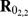
Simulation value	500.0	14.00	0.800	750.0	29.00	1.400
Initial value	350.3	12.18	0.599	615.8	21.34	1.061
EM REML	511.9	18.11	0.747	842.6	29.10	1.590
NR REML	512.1	18.20	0.730	842.3	29.02	1.607
AI REML	512.1	18.20	0.730	842.3	29.02	1.607
BM REML	512.6	18.08	0.751	841.9	29.13	1.586

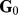
) and three unique residual (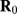) (co)variance components. All values are presented in thousands. The model includes three unique genetic (

All the algorithms used for analyzing the data were implemented in R software [21]. First we applied analytical EM REML, NR REML, AI REML and BM REML. For these analytical analyses we used convergence criteria based on relative squared changes in consecutive estimates, with 10^−10^ as the critical value. Each MC REML algorithm was tested with 20, 100 and 1000 MC samples per REML iteration round. The MC EM REML algorithm with 20 MC samples per REML round was used as a reference [4]. Estimates from round 2 of MC EM REML analysis were set as initial values for the Newton-type analyses (Table 1). For cases where Newton-type algorithms yielded estimates outside the parameter space, crash recovery was implemented by weighting the Newton-type and EM REML estimates with a weighting factor sequentially from 0.1 to 1.0 by 0.1 until the estimated VC matrices were positive-definite [15].

Convergence of an MC algorithm is difficult to identify, and so we examined the convergence performance of MC REML algorithms by continuing an additional 10 REML rounds more than required by corresponding analytical analyses. The obtained mean and relative standard deviations of the parameter estimates over the additional REML rounds are shown in Table 2. Three convergence criteria presented in the literature were then calculated for the MC AI REML algorithm. The first is a commonly used criterion, presented by Booth and Hobert [22], which is based on a change in consecutive parameter estimates relative to their standard errors. A value of 0.005 can be used as the critical value. The second criterion, by Kuk and Cheng [12], relies on the gradient vector and its variance-covariance matrix. Their stopping criterion is 90-percent quantile of a chi-square distribution with the number of parameters as degrees of freedom. This criterion attempts to stop the iteration as soon as possible. Finally, from MC AI REML round 5 onwards, convergence was also checked by a method similar to the one in Matilainen et al. [4], where the approach was to predict the parameter estimates of the next round using linear regression on previous iteration rounds. Here we took the same approach but applied the prediction method to the gradients instead of the estimates. Analyses were continued until the critical value of 10^−10^ as a norm for predicted round-to-round change in the gradient was reached.

**Table 2:** Means (relative standard deviation) of estimates over the last 10 rounds by MC REML.

Method	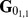	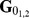	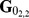	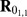	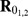	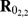
EM 20	519.3 (0.5%)	18.30 (0.5%)	0.752 (0.4%)	843.3 (1.1%)	28.98 (1.0%)	1.578 (1.0%)
NR 20	446.8 (60.8%)	15.54 (71.8%)	0.653 (67.5%)	877.3 (32.4%)	30.70 (35.4%)	1.655 (25.3%)
NR 100	509.8 (5.4%)	17.91 (6.4%)	0.712 (7.7%)	842.3 (2.6%)	29.20 (3.4%)	1.620 (3.3%)
NR 1000	510.9 (1.6%)	18.18 (2.1%)	0.730 (2.5%)	843.3 (0.8%)	29.04 (1.0%)	1.607 (0.9%)
AI 20	495.5 (7.2%)	17.44 (8.1%)	0.689 (8.4%)	855.3 (3.4%)	29.57 (4.5%)	1.632 (4.0%)
AI 100	513.4 (4.2%)	18.20 (4.7%)	0.729 (5.2%)	839.9 (2.6%)	28.93 (2.8%)	1.602 (2.4%)
AI 1000	513.8 (1.6%)	18.28 (1.9%)	0.734 (1.9%)	840.3 (0.9%)	28.92 (1.1%)	1.603 (0.8%)
BM 1000	502.1 (3.2%)	17.73 (3.5%)	0.758 (1.1%)	852.7 (1.9%)	29.48 (1.9%)	1.581 (0.5%)

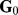
) and three unique residual (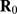) (co)variance components. Values were calculated over REML rounds 402 to 411 for MC EM REML, 6 to 15 for MC NR and MC AI REML, and 12 to 21 for MC BM REML with 20, 100 or 1000 MC samples. Mean values are presented in thousands. The model includes three unique genetic (

## Results

Analytical EM REML converged in 401 rounds, analytical NR REML and AI REML in 5 rounds, and analytical BM REML in 11 rounds. Estimates by analytical algorithms differed by less than 3% across algorithms, as seen in Table 1. The mean and relative standard deviation for the MC REML estimates obtained from the additional 10 REML rounds after reaching the convergence point determined by corresponding analytical algorithms are given in Table 2. Due to convergence problems in MC BM REML, only results with 1000 MC samples per REML rounds are reported here(Table 2). Almost all VC estimates were in good agreement with the analytical estimates, their means deviating less than 2.5% from the analytical ones. The exceptions were estimates by MC NR REML with 20 samples and those for genetic effect by MC AI REML with 20 samples. The variability of the estimates can be seen in the relative standard deviations over the last 10 REML rounds. Round-to-round variation in the MC EM REML estimates after assumed convergence was only 0.5% for genetic VC, while MC NR REML and MC AI REML with 100 samples per REML round would still have relative standard errors of 5%–8% and 4%–5%, respectively, in the corresponding estimates.

**Figure 1:**
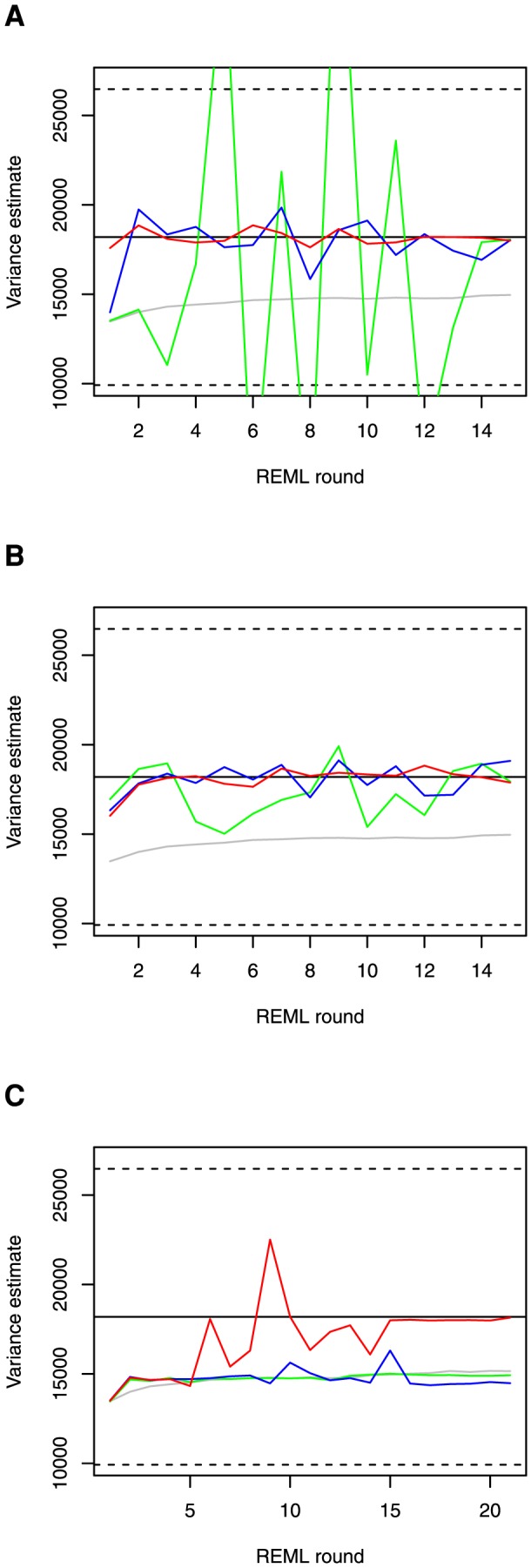
Estimates of the genetic covariance component by Newton-type methods. Analyses by MC NR REML (Figure A), MC AI REML (Figure B) and MC BM REML (Figure C) with 20, 100 and 1000 MC samples (green, blue and red line, respectively). MC EM REML with 20 MC samples is plotted as a reference (grey line). The straight lines in the figures are the estimated genetic covariance (solid line) and plus/minus one standard deviation (dashed lines) based on standard errors by analytical AI REML.

**Figure 2:**
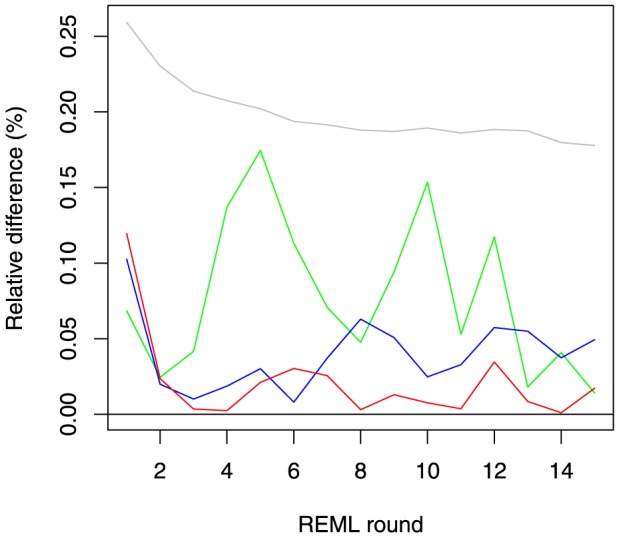
Relative difference between MC AI REML estimates and the true estimate obtained by analytical AI REML. The relative difference (%) is plotted for MC AI REML estimates with 20, 100 and 1000 MC samples (green, blue and red line, respectively) along the iteration. MC EM REML with 20 MC samples is plotted as a reference (grey line).

MC REML round-to-round convergence in the genetic covariance component 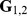 is illustrated in Fig. 1. The straight lines in the figures represent the estimated genetic covariance (solid line) and estimated standard error (dashed lines) by analytical AI REML. Fig. 2 describes the relative absolute difference between estimates obtained by MC AI REML with different numbers of MC samples and the true estimate by analytical AI REML.

The standard error of the genetic covariance estimates were 7996 and 8274 by the analytical NR REML and AI REML algorithms, respectively. Standard errors were not calculated by analytical EM REML and BM REML. When calculated by MC NR REML, standard errors for the genetic covariances at REML round 10 was 11360, 8056 and 8495 with 20, 100 and 1000 MC samples per round, respectively. Corresponding, standard errors by MC AI REML at REML round 10 were 8857, 8185 and 8294 with 20, 100 and 1000 MC samples per REML round. However, it should be noted that these actual numbers of standard errors may vary from round to round due to sampling.

Of the three different convergence criteria studied for the MC AI REML algorithm, the convergence criterion presented by Booth and Hobert [22] gave average values of 0.35, 0.15 and 0.05 with 20, 100 and 1000 MC samples per MC AI REML round, respectively. This indicates the need for a huge increase in MC sample size before the critical value of 0.005 proposed by Booth and Hobert [22] can be reached. Kuk and Cheng [12], in turn, suggest stopping the iteration at MC AI REML rounds 2, 1 and 1 with 20, 100 and 1000 MC samples per round, respectively. Their criterion implies relatively small gradients after 1 or 2 steps which is probably due to large standard errors of the estimates. According to the convergence criterion in Matilainen et al. [4] using a critical value of 10^−10^, iteration would stop at MC AI REML rounds 101, 70 and 44 with 20, 100 and 1000 MC samples per round, respectively. Because this criterion may be too strict for practical purposes in MC REML analyses, we also checked stopping at points when the criterion gave values less than 10^−8^. This would mean that analyses would be stopped at MC AI REML rounds 28, 27 and 10 with 20, 100 and 1000 MC samples, respectively.

## Discussion

Whereas the MC NR REML method is easy to implement, it may require a large number of MC samples to accurately approximate the variances of first derivatives over samples. MC AI REML, in contrast, works better even with small MC sample sizes, because the AI matrix has no extra sampling noise as it depends only on variance parameters estimated in the previous round. MC AI REML rounds are computationally more demanding than MC NR REML, however, because the MME system needs to be solved at each MC AI REML round as many times as there are VC parameters to be estimated.

MC BM REML is computationally the least expensive of the considered methods when the number of REML rounds and the number of MC samples are kept the same. To circumvent evaluation of the information matrix, BM REML corrects the approximation of the inverse of information matrix from round to round based on the gradients. While the analytical BM REML worked reasonably well, the small data set in our study required a large MC sample size for the method to work, which indicates its sensitivity to changes in gradients from round to round. Furthermore, MC BM REML is efficient even with a fairly poor approximation to the information matrix, but extra computations are needed for standard errors after convergence has been reached.

The performance of MC NR and MC AI REML was quite similar to analytical NR and AI REML. The only clear difference was that, with small MC sample sizes, estimates by MC NR REML varied more than those by MC AI REML. With 20 MC samples, the relative standard deviations from the last 10 REML rounds by both methods were unacceptably high, although MC AI REML was better. With 100 MC samples per REML round, the standard deviations were acceptable, and estimates by MC NR REML showed approximately as much variation as the estimates of MC AI REML. Thus, the information matrix appears to be quite accurately estimated in this case. With 1000 MC samples, variation in MC NR REML and MC AI REML estimates was almost equal. Genetic covariance estimates by both methods differed on average from the true value by 5% and 2% with 100 and 1000 MC samples, respectively. Interestingly, such variation diminished when MC BM REML was applied (Fig. 1c). Why this did not happen with MC NR or MC AI REML analysis may be because the diagonals in the approximation of the inverse of the information matrix were close to unity throughout the analysis, leading to more like MC EM REML parameter estimates.

For analytical REML analysis, Newton-type REML algorithms provide much faster convergence than EM REML, leading to shorter overall solving times with small data sets. The use of MC in the algorithms speeds up convergence of Newton-type methods, but sampling variation in the estimates increases compared to MC EM REML analysis. This is due to multiplication of the gradients by the inverse of the information matrix, as seen in the increase of MC noise. If each round of iteration in Newton-type methods requires many more samples than MC EM REML, overall solving time will reduced only in case the Newton method can enhance the convergence dramatically. The solving times were not recorded in this study because they would only apply to the model and implementation used. With respect to the total number of times to solve MME along the analysis, results showed that MC EM REML with 20 MC samples and 401 EM REML rounds corresponded to MC NR REML with 100 MC samples and 80 NR REML rounds or MC AI REML with 100 MC samples and 75 AI REML rounds. Thus, the number of times required to solve MME within a REML round is *s*+1 for MC NR REML but 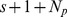 for MC AI REML, where *s* is the number of MC samples and 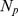 is the number of VC parameters. Hence, in our example with six parameters, MC NR and MC AI REML clearly outperformed MC EM REML, especially if we consider that the analytically implemented NR REML and AI REML needed 5 REML rounds to reach convergence but EM REML needed 401 rounds.

Obtaining a fast algorithm for REML estimation requires development of a practical convergence criterion for Newton-type methods. Although convergence is the same regardless of MC sample size, MC variation affects the values of the convergence criteria. Further study is therefore needed to define a suitable critical value for genetic evaluations. Identification of a feasible convergence criterion also requires deciding which values to use as the final solutions: the average of estimates over several REML rounds or simply the estimates at the last REML round.

The performance of MC-based algorithms is the better the larger the data to be analyzed. With a large data set, the averages of the gradients for MC AI REML are more accurate also with a smaller MC sample size, which leads to more accurate moves in the EM steps of MC BM REML. Most probably the amount of MC samples needed for sufficiently accurate gradient variances in MC NR REML will also decrease somewhat. As models grow larger and more complex, the efficiency of different methods becomes more difficult to predict. Further experience is especially needed on the behaviour of MC BM REML in VC estimation of complex models. A shortfall with respect to MC AI REML is that the number of times needed to solve MME increases along with increase in the number of estimated VCs. This fact does not change even with a large data set, and so MC NR and MC EM REML may become more efficient than MC AI REML. For instance, in [4], MC EM REML was used to estimate 96 VCs in a model describing daily milk yields of dairy cows. Estimation by MC EM REML with 5 MC samples per REML round required 565 rounds. The same analysis by MC AI REML with 20 MC samples per REML round should converge in less than 25 rounds to be computationally superior over MC EM REML, given that the MME solving time is the same for both algorithms.

The estimates of the analyses presented here were weighted by corresponding EM REML estimates whenever they fell outside the parameter space. Yet, this does not guarantee convergence to the true solutions, especially with respect to Broyden's method. To avoid divergence, Broyden [18] suggested choosing a scalar multiplier, i.e., a step length that decreases the change in some gradient norm and ensures the ascent of likelihood at each step. Convergence is also guaranteed by the Wolfe conditions [10], which ensure that steps make a sufficient ascent. However, if the search direction in BM REML approximates the Newton direction well enough, the unit step length will satisfy the Wolfe conditions, as the iterates converge to the solution [10]. Based on our study, this may mean that the required MC sample size may become enormous. One way to increase the robustness of VC estimation algorithms is reparametrization of the VC matrices by Cholesky decomposition [11]. The performance of this option is worth considering in future studies.

## Conclusions

Our results show that the use of MC algorithms in different Newton-type methods for VC estimation is feasible, although there was variation in efficiency between the implementations. An efficient MC method can achieve fast convergence and short computing times for VC estimation in complex linear mixed effects models when sampling techniques are used. However, analysis of our small simulated data implies that the number of MC samples needed for accurate estimation is dependent on the used method. This work encourages testing the performance of the presented methods in solving large-scale problems.
